# EPreNet: A Condition-Guided Network Accelerates Etching Profile Prediction

**DOI:** 10.3390/mi17050546

**Published:** 2026-04-29

**Authors:** Mengjiao Lu, Zerui Jin, Wanjun Wang, Jianghai He, Qingqing Liu

**Affiliations:** 1 School of Computer and Software Engineering, Anhui Institute of Information Technology, Wuhu 241199, China; lumengjiao@nnu.edu.cn (M.L.); wjwang@nnu.edu.cn (W.W.); jhhe10@iflytek.com (J.H.); 2 Institute of EduInfo Science and Technology, Nanjing Normal University, Nanjing 210024, China; 3 School of Electronic Science and Engineering, Nanjing University, Nanjing 210023, China; zerui_jin@smail.nju.edu.cn

**Keywords:** plasma etching, etching profile prediction, spatio-temporal modeling, semiconductor manufacturing, TCAD simulation

## Abstract

Plasma etching is a critical step in semiconductor manufacturing, yet existing approaches are either computationally expensive or limited to predicting scalar etching metrics rather than full profile evolution. We propose EPreNet, a condition-guided spatio-temporal network for pixel-level prediction of plasma etching profile evolution from historical profile frames and process parameters. To support this task, we construct a benchmark dataset of 18,360 images spanning 918 process conditions simulated via TCAD, sampled with Latin Hypercube Sampling to ensure uniform parameter-space coverage, and further establish an evaluation framework combining image-level and geometry-based metrics for etching-profile prediction. Experiments demonstrate that EPreNet reduces MSE by 16% and achieves SSIM of 0.992 and PSNR of 30.323 dB, while achieving manufacturing-relevant geometric accuracy with 1.4° sidewall angle error and 1.6% depth error rate. Inference requires only 38.92 ms per frame faster than TCAD simulation 1300 s, supporting rapid surrogate-based evaluation and accelerated TCAD-assisted process exploration. The model also shows strong generalization to unseen initial critical dimensions and encouraging initial transferability to preprocessed experimental SEM images, suggesting its potential as an efficient surrogate for TCAD-assisted process development while maintaining high geometric fidelity.

## 1. Introduction

Plasma etching constitutes a critical and indispensable process step in integrated circuit (IC) manufacturing, particularly for advanced technology nodes. Etching profile geometry directly determines key device functionalities, including inner spacer cavities in complementary field-effect transistors (CFETs), aspect ratios in fin field-effect transistors (FinFETs), and precise sidewall spacer angles, thereby profoundly impacting electrical performance and manufacturing yield [1]. As semiconductor device geometries continue to shrink toward 3 nm, and even more aggressive 2 nm nodes, etching processes face unprecedented challenges. Complex coupling among chamber pressure, RF power, gas flow rates, and surface chemistry creates a high-dimensional parameter space with strong nonlinearities, rendering process optimization computationally intensive. Traditional plasma etching process optimization has relied primarily on iterative experimental cycles, an approach that becomes prohibitively time-consuming and costly when targeting sub-nanometer dimensional control. This industrial imperative has driven the semiconductor community to seek computational modeling tools capable of accelerating process development while preserving sufficient fidelity for simulation-based process evaluation and accelerated development.

Physics-based Technology Computer-Aided Design (TCAD) simulations have emerged as a cornerstone for etching process modeling, providing high-fidelity predictions by modeling governing equations for charge transport, gas-phase chemistry, ion bombardment, and surface reactions. While TCAD shifts development from empirical trial-and-error to a model-experiment-validation workflow, it is time-consuming. Typical 3D feature-scale etching simulations consume approximately 6500 CPU seconds (roughly 1.8 h) per prediction [2]. Even with parallel computing infrastructure, comprehensive Design of Experiments (DoE) exploring hundreds of parameter combinations requires weeks of wall-clock time.

Recent deep learning approaches, such as physics informed neural networks (PINNs) [3] and data-driven methods [4,5,6], have been proposed to accelerate physics-based modeling by predicting etching depth and mask residual thickness, thereby improving development efficiency. In particular, works [4,5] leveraged sensor data to predict etching depth and mask residual thickness. However, such scalar regression methods inherently sacrifice spatial information, failing to capture critical geometric features like sidewall bowing, undercut profiles, or scalloping patterns that determine device performance. By contrast, image-based prediction approaches are able to capture spatially resolved profile geometry and compute multidimensional numerical metrics via image processing, enabling simultaneous monitoring and comprehensive quantitative evaluation of etching profile features. Xiao and Ni [6] proposed a multi-scale modeling framework combining recurrent neural networks for optimizing plasma etching processes. Guo et al. [2] introduced an attention-enhanced conditional variational autoencoder (CVAE) that integrates TCAD simulation priors with attention mechanisms, improving process efficiency. While the model improves process efficiency, it lacks sufficient interpretability, and effectively combining physical laws with data-driven deep learning remains an open challenge.

To address these limitations, we propose an etching profile prediction network—EPreNet, which enables pixel-level prediction of etching profile from historical image sequences and process parameters. Unlike hard PDE constraints used in PINNs, we embed process condition parameters into image features to achieve condition-guided etching-profile prediction. The network consists of (i) a spatial encoder with local window attention to extract fine-grained boundary features; (ii) a condition-guided feature module (CFM) that injects process parameters via learnable affine transformations; and (iii) a causal temporal module combining multi-layer convolutional long short term memory (LSTM) with causal temporal attention (CTA) to enforce temporal irreversibility. Additionally, we construct a benchmark dataset of 18,360 images (512 × 512) from 918 process conditions sampled with Latin Hypercube Sampling to ensure uniform parameter-space coverage. Sparse-frame prediction reduces the number of required simulation snapshots, shortening the process development cycle. Our contributions are summarized as follows:

- EPreNet enables a prediction-accelerated workflow for etching process development by partially replacing TCAD simulations, and model’s inference speed of 38.92 ms per frame is orders of magnitude faster than TCAD simulations (1300 s), making it suitable for surrogate-assisted screening and rapid process-space exploration.
- Three physics-based geometric metrics (EDE, SAE, and BCDE) are proposed and combined with image-quality metrics (MSE, SSIM, and PSNR) to form a comprehensive evaluation protocol for etching profile prediction, addressing the limitation of prior work that relies solely on image-level assessment.
- EPreNet achieves SSIM > 0.992, PSNR > 30 dB, and 16% reduction in MSE compared to other prediction methods, while maintaining high profile-level geometric fidelity (0.032 μm EDE, 1.4° SAE and 0.029 μm BCDE), and it also demonstrates strong generalization to unseen initial-CD geometries under fixed process conditions, and attains an average SSIM of 0.93 on preprocessed experimental SEM images.

## 2. Related Work

### 2.1. Plasma Etching Simulation

Plasma etching is a fundamental step in semiconductor manufacturing, involving coupled phenomena such as ion bombardment, chemical reactions, and species transport. Process parameters—including chamber pressure, RF power, and gas flow rates—are highly nonlinear and interdependent, creating a high-dimensional parameter space with strong nonlinear interdependencies that directly affects device geometry, performance, and yield [7,8]. Simulation tools are therefore essential to understand parameter-process relationships before fabrication.

TCAD simulations achieve high-fidelity etch profile predictions by numerically solving equations for charge transport, gas-phase chemistry, and surface reactions [9]. Monte Carlo methods, widely used for particle-level modeling, offer high accuracy but require several hours to days per simulation [10]. While TCAD captures anisotropy, sidewall morphology, and microloading effects, the computational cost limits rapid exploration and real-time optimization, highlighting a trade-off between accuracy and efficiency.

### 2.2. Deep Learning for Etching Processes

The rapid development of deep learning has greatly expanded its application in semiconductor manufacturing, leading to significant improvements in device development efficiency and yield [11]. Existing applications broadly fall into three categories. The first involves feature extraction and process optimization, leveraging nonlinear modeling and interpretability tools to adjust etching parameters, such as [5,12]. Kanarik et al. [1] proposed a human-machine collaborative framework leveraging Bayesian optimization, reducing experimental cost by approximately half for recipe determination. The second direction focuses on process monitoring and anomaly detection, using sensor data or image-based models to identify equipment drift, process deviations, and proximity-induced variations prior to fabrication [13,14,15], thus enabling design corrections prior to manufacturing. The third category—etching profile prediction—aims to forecast evolving etching state from process parameters or historical measurements using numerical or image-sequence inputs. Early approaches focused on predicting individual etching metrics, e.g., Chen [16] used deep neural networks to predict etching bias for 2D patterns with an average relative error below 15%, while Xiao and Ni [6] developed a multi-scale RNN-based framework for 3D plasma etching optimization.

Despite these advancements, most models are limited to key parameters such as depth, roughness, or critical dimensions, leaving full profile prediction largely unexplored. Since each pixel in a cross-sectional etching profile evolves over time, complete profile sequences can be naturally viewed as spatio-temporal data, motivating the adoption of sequence prediction methods. Methods for multivariate time series prediction using RNNs and graph neural networks have shown success in related domains [17,18,19,20], providing methodological foundations for the approach proposed in this work.

## 3. Problem Formulation and Dataset

### 3.1. Problem Formulation

Etching profile evolution prediction is formulated as a condition-based spatio-temporal forecasting problem. Given the observed sequence $X=\left\{x_{1},x_{2},\ldots ,x_{i}\right\}\in R^{T\times C\times H\times W}$ (where *T* denotes time steps, *C* means channel of images, and *H* and *W* represent image height and width, respectively) and process condition vector $\theta \in R^{n}$ (where *n* is the number of process parameters), the goal is to predict etching profile evolution $\hat{Y}$ in future $t^{\prime }$ steps:

(1) $$\hat{Y}=\left\{{\hat{y}}_{T+1},\ldots ,{\hat{y}}_{T^{\prime }}\right\}$$

where $T^{\prime }=T+t^{\prime }$ denotes the full etching duration, and each frame ${\hat{y}}_{i}\in R^{C\times H\times W}$ represents the etching profile image at a certain moment. In summary, the goal is to learn a mapping function $f:\left(X,\theta \right)\mapsto \hat{Y}$ that can accurately predict the future etching profile evolution based on historical image sequence and process conditions.

### 3.2. Proposed Dataset

#### 3.2.1. TCAD Simulation Setup

Plasma etching involves complex interactions among numerous process parameters [21]. Variations in chamber pressure significantly affect the ion mean free path and energy distribution, thereby influencing etching anisotropy and sidewall profiles [22]. An increase in substrate bias has been correlated with changes in etching rate and sidewall morphology [23], and argon flow rate strongly affects profile [24]. Based on these sensitivities, five critical parameters with the greatest impact on profile morphology are selected as the process condition vector θ, including chamber pressure (*P*), top electrode RF power ($P_{\mathrm{top}}$), bottom electrode RF power ($P_{\mathrm{bot}}$), Ar flow rate ($Q_{\mathrm{Ar}}$), and ${\mathrm{CF}}_{4}$ flow rate ($Q_{{CF}_{4}}$). Latin Hypercube Sampling (LHS) is employed to generate 918 parameter combinations, ensuring uniform coverage of the high-dimensional parameter space while avoiding the combinatorial explosion inherent in traditional grid sampling. The ranges, step sizes, and units of these parameters are summarized in Table 1.

Notably, we employ Sentaurus TCAD 2022.03 to simulate the etching process. Simulations are conducted on a Linux system (CentOS 7) equipped with a 64 GB Intel Xeon Gold 6226R CPU (2.90 GHz), requiring approximately 1300 s per simulation.

#### 3.2.2. Data Acquisition and Preprocessing

**Figure 1:**
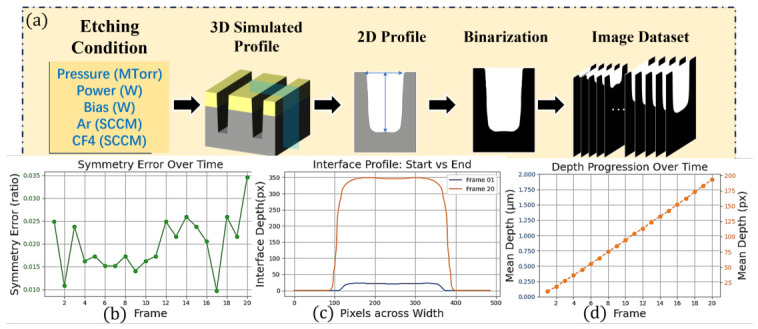
Data collection process and visualization. (**a**) Data acquisition and preprocessing pipeline. (**b**–**d**) show unique properties of etching profile sequences.

A 2×2μm 2D cross-sectional domain is simulated to capture microloading effects and local transport limitations in trench etching. For 918 parameter combinations, each simulation runs for 600 s with snapshots recorded every 30 s, yielding 20 temporal frames per condition. The total computational cost is 331.5 h (≈2 weeks), highlighting the computational bottleneck of TCAD, which motivates the use of our deep learning surrogate. As shown in Figure 1a, 2D cross-sections along the x-z plane are extracted, binary segmented—where unetched ${\mathrm{SiO}}_{2}$ and etched regions are used as background and foreground, respectively—and resized to 512×512 pixels. The final dataset contains 18,360 images (918 sets × 20 frames), partitioned into training (734 sets, 80%, 14,680 images) and test sets (184 sets, 20%, 3680 images), ensuring evaluation on entirely unseen parameter combinations and objectively assessing generalization across the etching parameter space.

### 3.3. Etching Profile Sequence Attributes

Constructed etching profile sequences exhibit two intrinsic characteristics that distinguish them from generic spatio-temporal forecasting or natural video sequences (Figure 1b–d), providing explicit design guidelines for the task-specific EPreNet architecture. (1) Structural sparsity. Information is concentrated on the boundaries of etching profiles, while static regions maintain high spatial homogeneity, with symmetry error remaining below 0.035 across all frames (Figure 1b), confirming the high spatial homogeneity of static regions. Etching occurs only at the substrate boundary, and previously etched regions remain unchanged (Figure 1c). Computing global self-attention over the full image wastes computation on these static regions, motivating the adoption of local window attention to improve computational efficiency. (2) Monotonic causal evolution. Etching depth increases monotonically at an approximately linear rate of 1.2±0.3nm/s across all process conditions without reversal (Figure 1d). This necessitates causal temporal modeling, where future predictions ${\hat{y}}_{i}$ depend on historical observations $\left\{x_{1},\ldots ,x_{i-1}\right\}$ and process conditions θ.

## 4. Architecture of Network

**Figure 2:**
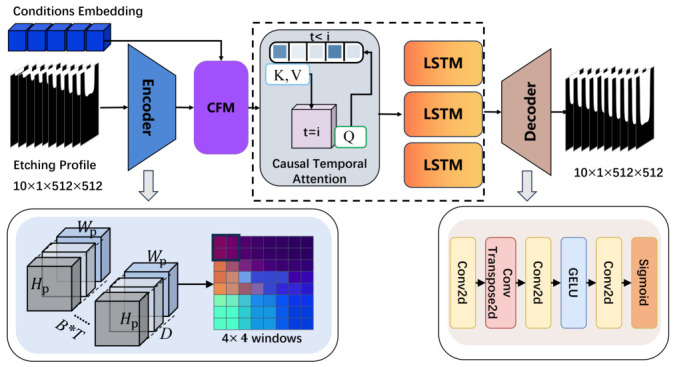
Overview of our EPreNet.

As illustrated in Figure 2, EPreNet takes two inputs: a sequence $X\in R^{T\times C\times H\times W}$ (where C=1, H=W=512), and process parameter vector (1) Spatial Encoding. Patch embedding with local window attention efficiently captures fine-grained boundary features of etching profiles while avoiding the computational overhead of global attention. (2) Process Conditioning. CFM modulates image features based on θ via learnable affine transformations, producing condition-aware features. (3) Temporal Modeling. Multi-layer causal LSTM combined with causal temporal attention (CTA) propagates temporal dependencies while enforcing future information masking. Finally, decoder reconstructs predicted etching profiles from temporally processed features.

### 4.1. Spatial Feature Extraction

Spatial feature extraction is designed to capture fine-grained geometric and boundary features of etching profiles while maintaining computational tractability for high-resolution images. Self-attention in standard vision transformers (ViT) [25,26] incurs prohibitive computational costs for high-resolution images because its complexity scales quadratically with the number of patches. Thus, we introduce a patch-based spatial encoder integrating patch embedding with local refinement and window attention.

#### 4.1.1. Patch Embedding with Local Refinement

Given a batch of past frames $X\in R^{B\times T\times C\times H\times W}$, where *B* denotes batch size, we reshape the input tensors into tensors of shape (B×T)×C×H×W, then use convolution to split each image into non-overlapping patches:

(2) $$Z_{t}={Conv}_{p\times p}\left(x_{i}\right),i\in \left\{1,2,\ldots ,T\right\}$$

where Conv(·) means convolution, and *p* is the patch and convolution kernel size, balancing feature granularity and computational efficiency. $h_{p}=H/p$, $w_{p}=W/p$ denote the patch feature grid height and width. To mitigate information loss from downsampling, initial patch embeddings are refined through two 3×3 convolutional layers:

(3) $$Z_{t}^{f}=2\cdot GELU\left({\mathrm{Conv}}_{3\times 3}\left({\mathrm{Conv}}_{3\times 3}\left(Z_{t}\right)\right)\right)$$

where GELU(·) is the activation function, ensuring stable propagation across layers. This design captures the intra-patch texture gradients and edge sharpness critical for precise etching front localization.

#### 4.1.2. Window Attention

Capturing spatial dependencies across distant trench regions—such as the influence of top trench openings on bottom etching rates via plasma transport and ion bombardment [27]—is critical for accurate modeling. However, applying global self-attention across entire patch grid incurs computational complexity of $O(h_{p}^{2}w_{p}^{2}D)$, making it computationally inefficient for sparse etching profiles. Local window attention is adopted to efficiently model within-window spatial dependencies, while the shifted-window mechanism of the Swin Transformer [28] enables cross-window information exchange across the full feature map. Feature $Z_{t}^{f}$ is divided into local windows of size w×w, within which self-attention is performed, reducing complexity to $O(h_{p}w_{p}\cdot w^{2}D)$.

### 4.2. Condition-Guided Feature Module (CFM)

#### 4.2.1. Architecture of CFM

Standard approaches for injecting conditioning information—such as feature concatenation or additive fusion—treat all process parameters uniformly and fail to capture the nonlinear, channel-selective modulations associated with physically meaningful differences among process conditions. Inspired by feature-wise linear modulation [29], the CFM injects process condition information θ into the spatial feature map $Z_{t}^{f}$ in a channel-wise manner. The modulated feature is computed as follows:

(4) $$Z_{t}^{c}=\left(1+\gamma (\theta )\right)⊙Z_{t}^{f}+\beta \left(\theta \right)$$

where γ(θ) and β(θ) are channel-wise scaling and bias factors, respectively, and ⊙ denotes element-wise multiplication broadcast across spatial dimensions. Modulation parameters are derived from θ via a learnable embedding and linear projection:

(5) $$v=\mathrm{Linear}\left(\mathrm{Flatten}(\mathrm{Embed}(\theta ))\right)$$

where Embed(·) is a learnable embedding layer, and Linear(·) adjusts feature channels. Feature *v* is split along the channel dimension to yield the raw scaling factor $\gamma_{0}\left(\theta \right)$ and the bias term β(θ), where $\gamma_{0}$ is further processed via Equation (6) to ensure stable training and physically meaningful modulation. $\gamma_{0}\left(\theta \right)$ is passed through a sigmoid function and scaled by a learnable scalar *s*:

(6) $$\gamma \left(\theta \right)=s\cdot \sigma (\gamma_{0}\left(\theta \right))$$

where σ is the sigmoid function and constrains the modulation to [0,1], and *s* controls the maximum modulation strength across channels.

#### 4.2.2. Interpretability Analysis of Condition-Guided Modulation

**Figure 3:**
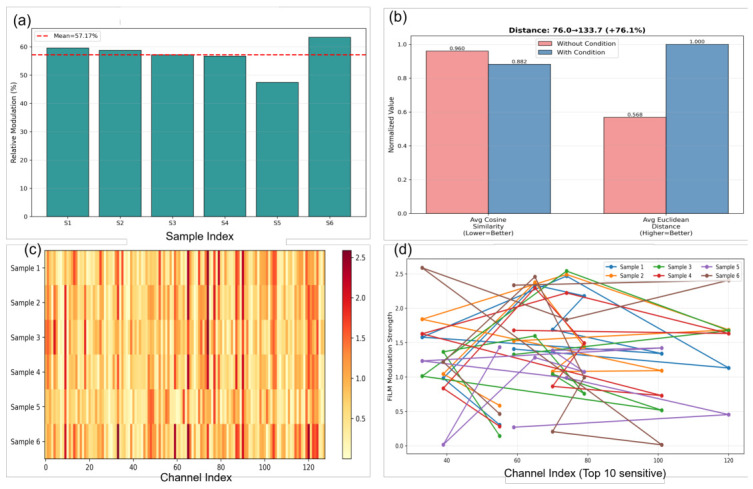
Visualization of CFM modulation patterns under varying process conditions. (**a**) Average modulation strength across all samples. (**b**) Cosine similarity comparison between CFM and w/o CFM. (**c**) Channel-wise modulation patterns for six process parameter variants. (**d**) Top-10 most condition-sensitive channels.

**Figure 4:**
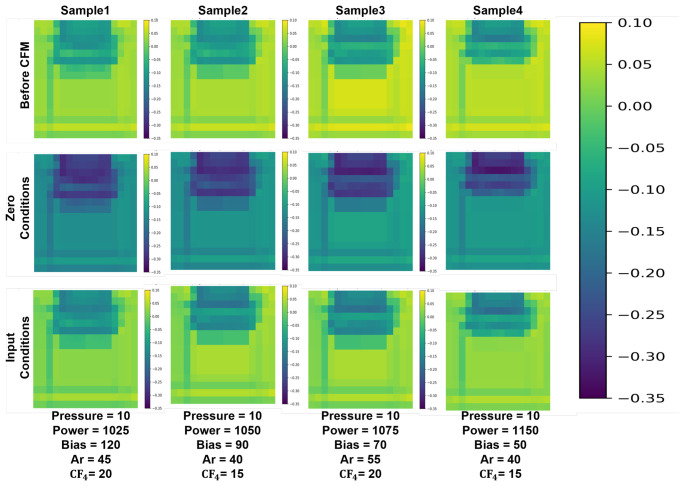
Channel-wise CFM modulation patterns under different process variants, visualization of heatmaps before and after condition injection.

To validate the design of CFM, we analyze feature modulation behaviors under six distinct process parameter variants (Table 2), evaluating global modulation strength and channel-wise activation patterns as visualized in Figure 3. Feature modulation intensity is quantified using the $l_{2}$ norm of the difference between conditioned and unconditioned features. As shown in Figure 3a, the average modulation strength across all samples is 57.17%, ranging from 47.44% to 63.37%, indicating balanced allocation between spatial features and process-dependent conditioning. To verify condition-specific feature transformations, we compute the cosine similarity and Euclidean distance. Compared with the CFM-free model, CFM reduces cosine similarity by 8.1% and increases the Euclidean distance by 76.1% (Figure 3b and Figure 4).

Channel-wise modulation patterns in Figure 3c show that changes in different process parameters selectively activate distinct channels. For instance, Sample 1 (elevated bottom power) strongly activates channels 0 to 40, Sample 3 (doubled ${\mathrm{CF}}_{4}$ flow) exhibits a prominent peak around channel 75, and Sample 5 (reduced top power) displays relatively weak overall modulation. Analysis of the top-10 most condition-sensitive channels in Figure 3d reveals correspondence between channel indices and dominant physical factors: lower-indexed channels predominantly respond to variations in bottom power, mid-indexed channels to ${\mathrm{CF}}_{4}$ flow rate, and higher-indexed channels to chamber pressure. These patterns suggest that the CFM module produces interpretable condition-dependent modulation and that the learned feature variations are broadly consistent with known process trends.

## 5. Experiments

### 5.1. Implementation Details

The proposed EPreNet is implemented in the PyTorch 2.0.0 framework and trained on a single NVIDIA RTX 4090 GPU. We use Adam optimizer [30] with an initial learning rate of $1\times 10^{-4}$ and weight decay of $5\times 10^{-6}$. All etching images are resized to 512×512 pixels. The batch size is set to 4, and the model is trained for 100 epochs. A OneCycleLR learning rate scheduler is employed, with the maximum learning rate set to 10 times the initial value to stabilize convergence. The model takes 10 historical frames as input and predicts the subsequent 10 frames.

### 5.2. Evaluation Metrics

**Figure 5:**
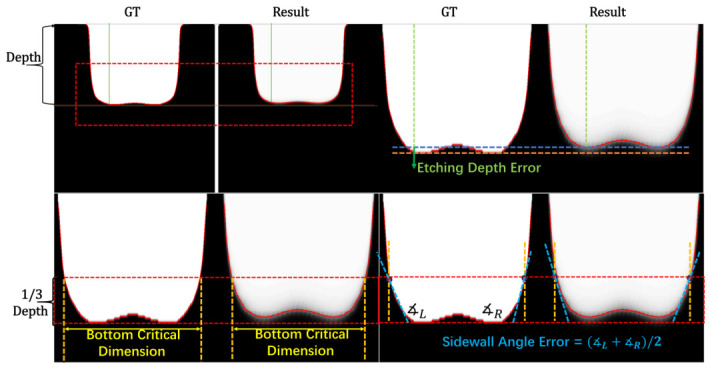
Illustration of physics-based metrics calculation.

Prediction performance is evaluated using both image-level metrics and geometry-based metrics. MSE, SSIM, and PSNR quantify pixel-wise fidelity and structural similarity between the predicted and ground-truth profiles. However, because etching-profile prediction is ultimately concerned with contour evolution rather than natural-image realism, image-level metrics alone are insufficient to characterize local geometric deviations. We therefore introduce three geometry-based metrics (EDE, SAE, and BCDE) to evaluate profile-level geometric fidelity, which metrics provide physically interpretable descriptions of morphology prediction accuracy (Figure 5).

The Etching Depth Error (EDE) is the signed difference between predicted and ground-truth etching depths. Etching depth is defined as the vertical distance between the top boundary of the etched region and its effective bottom boundary. Given the foreground pixel set,

(7) $$\Omega =\{\left(a_{i},b_{i}\right)|I\left(a_{i},b_{i}\right)=1\}$$

where $a_{i}$ is the x-coordinate and $b_{i}$ is the y-coordinate. The top position is computed as the minimum row coordinate, while the bottom position is estimated by the approximate maximum of foreground row coordinates to suppress the influence of isolated noise pixels. The etching depth is given by $b_{\mathrm{bot}}-b_{\mathrm{top}}$.

The Sidewall Angle Error (SAE) is the signed difference between predicted and ground-truth sidewall angles. The sidewall angle is estimated from the lower one-third of the etching depth. For each row within the fitting window, the leftmost and rightmost foreground pixels are extracted to form the left and right sidewall point sets. Linear regression is applied independently to each set, and the sidewall angle relative to the vertical direction is computed from the regression slope *m* as

(8) $$\theta =90^{\circ }-\arctan\;\left(\frac{1}{\left|m\right|}\right)$$

and the final anisotropy index is defined as the average of two sidewall angles.

The Bottom Critical Dimension Error (BCDE) is the signed difference between the predicted and ground-truth trench widths at the bottom. The bottom region is defined as the lower one-third of the etching depth, i.e., rows within $\left[b_{\mathrm{bot}}-\frac{1}{3}D,\;b_{\mathrm{bot}}\right]$. This threshold isolates the flat bottom plateau from the tapered sidewall transition zone above it. For each valid row in this region, the leftmost and rightmost foreground pixels are identified, and the bottom width is computed as the average horizontal span across these rows:

(9) $$\left\{\begin{array}{c} a_{\min}\left(b\right)=\min\left\{a|\left(a,b\right)\in R_{b}\right\},\\ a_{\max}\left(b\right)=\max\left\{a|\left(a,b\right)\in R_{b}\right\},\\ BCDE=\frac{1}{|B_{b}|}\sum_{b\in B_{b}}\left(a_{\max}\left(b\right)-a_{\min}\left(b\right)\right) \end{array}\right.$$

where $R_{b}$ denotes the set of all foreground pixels within the lower one-third region of trench, and $B_{b}$ denotes the set of valid rows in this bottom region.

EDE and BCDE are reported in pixels (px) and can be converted to micrometers (μm) via a calibrated pixel-to-physical-length scaling factor, where 1 px corresponds to approximately 0.0019 μm in the simulated 2×2μm domain. Inference efficiency is also measured by inference time (ms) and frames per second (FPS).

### 5.3. Comparative Performance Analysis

We compare EPreNet against five representative spatio-temporal prediction architectures, including ConvLSTM [31], PredRNN [32], PhyDNet [33], SimVP [34], and SimVPv2 [35]. All baselines are trained on the same dataset with 10 frames configurations and undergo hyperparameter tuning specifically for the etching task to ensure fair comparison.

#### 5.3.1. Quantitative Comparison

Table 3 summarizes the quantitative performance of all compared methods. EPreNet achieves the best overall image-level performance, reducing MSE by 16.03% relative to SimVP [34], while attaining the highest SSIM (0.992) and PSNR (30.323 dB). In terms of inference speed, EPreNet runs at 102.78 FPS, significantly faster than competing methods, demonstrating high inference efficiency for surrogate etching-profile prediction. Although EPreNet does not obtain the absolute lowest values on all physics-based metrics, it effectively controls the EDE rate and BCDE rate to 1.61% and 1.47%, respectively. Overall, EPreNet delivers the most balanced performance across image quality, physical accuracy, and inference efficiency—three dimensions that are jointly relevant to semiconductor process development.

#### 5.3.2. Qualitative Comparison

**Figure 6:**
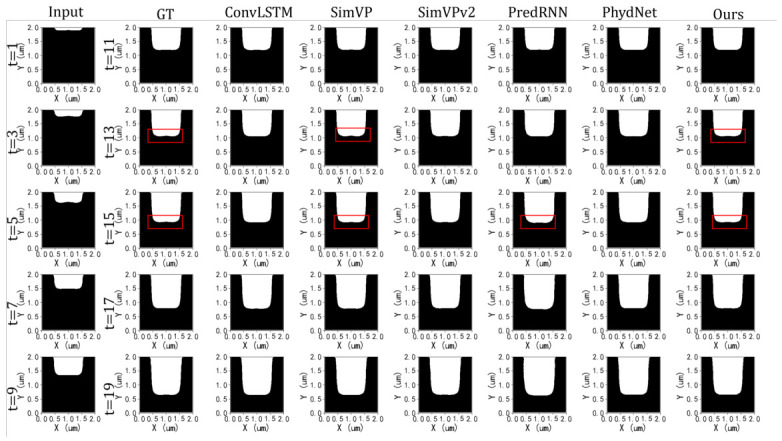
Visual comparison of EPreNet with other methods.

Figure 6 shows the qualitative results. Our EPreNet and SimVP [34] preserve the sharp boundaries and irregular profile geometry, whereas ConvLSTM [31] and SimVPv2 [35] exhibit over-smoothed structures. PhyDNet occasionally shows oscillations, and PredRNN [32] presents significant structural distortions, including sidewall bowing deviations from ground truth.

### 5.4. Ablation Study

To assess the contribution of each architectural component, we conduct ablation experiments by removing or replacing individual modules while keeping other settings. Results are summarized in Table 4 and Table 5.

#### 5.4.1. Effectiveness of Modules

Removing the local window attention increases MSE by 34.57% and decreases PSNR by 1.1 dB in Table 4 (a), demonstrating its crucial role in capturing structured spatial context and cross-region feature interactions. A drop in PSNR indicates that structured spatial context modeling is essential for preserving fine-grained boundary details, directly impacting geometric feature extraction such as sidewall angle and bottom critical dimension.

Removing CFM increases MSE by 30.42% and reduces PSNR by 1.31 dB in Table 4 (b). This performance degradation validates that integrating process condition information into feature modulation is essential for accurate etching profile evolution modeling and interpretability.

Temporal modeling captures the irreversible, long-term evolution of profiles. As shown in Table 4 (c), removing LSTM leads to dramatic degradation, 1000 × MSE rises to 1.494, PSNR drops by 24.01%, and SSIM decreases by 2.12%. This highlights the indispensable role of LSTM in propagating long-range temporal dependencies across sequential frames. Removing CTA increases MSE by 24.9% and decreases PSNR by 3.63%, showing that CTA refines temporal relationships while maintaining causal consistency with physical constraints.

#### 5.4.2. Loss Function

Using BCE loss alone results in the poorest performance, over-emphasizing foreground–background separation while neglecting geometric fidelity. As shown in Table 4 (f), the combination of MSE + SSIM results in a 10.5% increase in MSE compared to the full EPreNet loss function. This indicates that BCE still contributes to the overall performance gain, even with a relatively low loss weight of $\alpha_{1}=0.1$.

#### 5.4.3. Input Frame Length

**Figure 7:**
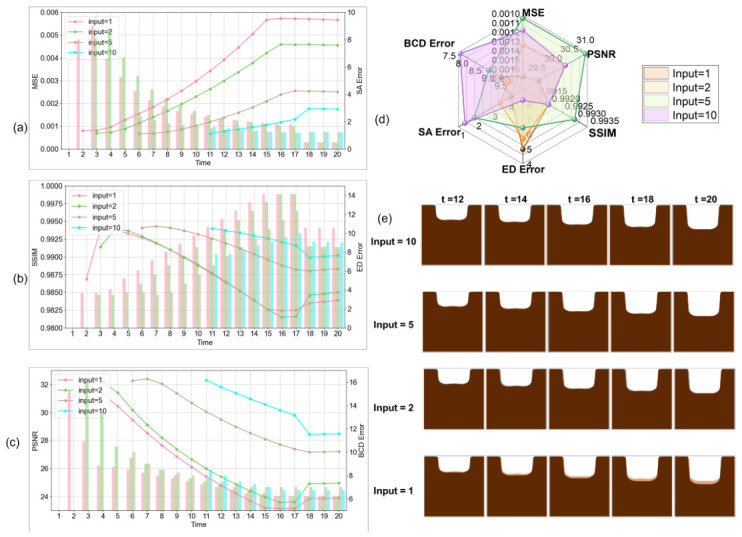
Visualization of predictions for different input frame lengths. (**a**–**c**) Per-frame metrics for input lengths 1, 2, 5, and 10. (**d**) Average metrics for the first 10 predicted frames. (**e**) Predicted frames 11–20 with ground truth (GT) profiles.

Figure 7 and Table 5 shows per-frame performance across different input lengths (1, 2, 5, and 10 frames). Early prediction is feasible with 1–2 frames but long-term accuracy deteriorates. Figure 7d,e summarize the average metrics and visualize predicted frames 11–20 compared with the ground truth.

Notably, EDE does not monotonically decrease with more input frames; with 10 input frames, EDE (8.28 px) is higher than that with 2 frames (6.74 px). This may reflect a trade-off, where longer input sequences improve overall image-level fidelity (lower MSE, higher SSIM/PSNR) while increasing the complexity of depth boundary localization.

### 5.5. Generalization Study

#### 5.5.1. Generalization to Unseen Initial Geometries

**Figure 8:**
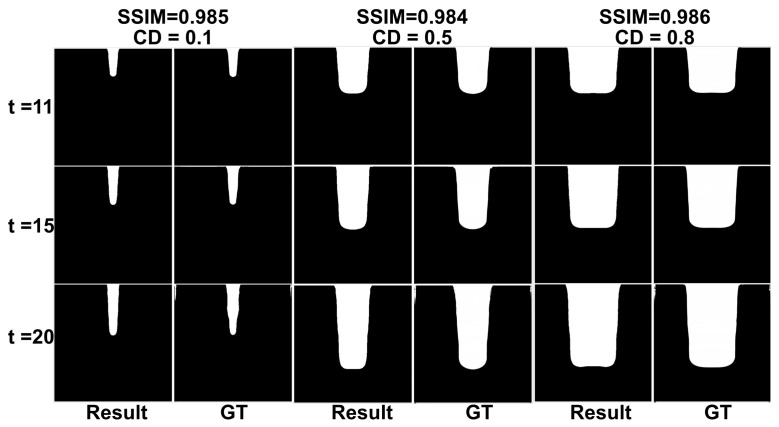
Qualitative comparison of EPreNet with different CDs.

To assess geometric transferability, we conduct a generalization experiment in which the initial trench critical dimension (CD) is varied from 0.1 to 0.99 μm while all process parameters are held fixed at their nominal values. This setting is intended to evaluate whether a model trained under one nominal geometry can still reproduce etching-profile evolution for unseen initial opening sizes within the same process family. As shown in Figure 8 and Table 6, EPreNet maintains SSIM above 0.98 across all CD variants, with an average EDE of 8.22 px (error rate 1.61%) and an average BCDE of 7.07 px (error rate 1.38%), indicating consistent profile-level geometric fidelity under unseen CD conditions. It is worth noting that the angle-related metric becomes less stable for very small CDs (e.g., 0.1 μm), where the limited number of foreground pixels in the measurement region reduces the stability of linear-regression-based angle estimation.

#### 5.5.2. Generalization to Experimental SEM Images

Assessing applicability beyond TCAD-generated data, we apply EPreNet directly to SEM cross-sectional image sequences acquired from actual plasma etching experiments, without any fine-tuning. SEM images are first binarized, and the first 5 frames of each sequence are used as input to predict the subsequent frames. Two representative process conditions are evaluated: (i) P=6 mTorr, $P_{\mathrm{top}}=1000$ W, $P_{\mathrm{bot}}=100$ W, $Q_{\mathrm{Ar}}=40$ sccm, $Q_{{\mathrm{CF}}_{4}}=20$ sccm; and (ii) P=6 mTorr, $P_{\mathrm{top}}=500$ W, $P_{\mathrm{bot}}=100$ W, $Q_{\mathrm{Ar}}=40$ sccm, $Q_{{\mathrm{CF}}_{4}}=20$ sccm. As shown in Table 7, EPreNet achieves an average SSIM above 0.930; although these values are lower than those on the TCAD-generated test data, EPreNet also reproduces the main etch-front progression and sidewall morphology observed in the SEM ground truth for both conditions.

Generalization experiments demonstrate that EPreNet exhibits geometric transferability to unseen initial CD configurations (SSIM > 0.98) and encouraging cross-domain transferability to experimental SEM data (SSIM ≈ 0.93), without retraining. These results provide initial evidence that the model can transfer from TCAD-generated data to preprocessed real SEM sequences under limited experimental conditions, although broader validation remains beyond the scope of the present study.

## 6. Conclusions

Experiments demonstrate that EPreNet effectively predicts plasma etching profile evolution with high accuracy and computational efficiency. Across the 918 conditions, the model consistently achieves superior performance compared to benchmark spatio-temporal architectures, with 16% reduction in MSE, SSIM exceeding 0.992, and a PSNR of 30.323 dB. These quantitative improvements suggest the model’s ability to capture fine-grained details, sidewall angles, and bottom critical dimensions. In addition to accuracy, EPreNet reduces inference time to 38.92 ms per frame, enabling rapid profile prediction for TCAD-assisted workflow acceleration and process optimization. Generalization experiments further demonstrate the model’s geometric generalization capability, which across nine unseen initial CD configurations ranging from 0.1 to 0.9 μm, SSIM consistently exceeds 0.98, and direct application to experimental SEM images without fine-tuning yields an average SSIM of 0.93, providing initial evidence of transferability from TCAD-generated data to preprocessed real SEM sequences. EPreNet combines local window attention, CFM, LSTM and CTA to enforce task-specific inductive biases, producing condition-aware and geometrically consistent etching predictions. In particular, CFM demonstrates that selective channel activation is aligned with process parameters, suggesting that the learned feature modulations are consistent with known process trends related to RF power, ${\mathrm{CF}}_{4}$ flow and pressure. Feature discrimination analysis further shows that condition-dependent embeddings increase Euclidean distance, verifying the model’s capacity to differentiate between distinct process regimes.

In summary, these results indicate EPreNet as an accurate, efficient, and interpretable alternative for plasma etching process development, capable of accelerated TCAD-surrogate screening workflows while maintaining the geometric fidelity.

Despite these encouraging results, several limitations of the present study should be acknowledged. First, the model is trained on TCAD-generated profile images, which contributes to the simulation-to-reality gap observed in the SEM transfer experiments. Second, the current dataset is confined to ${\mathrm{SiO}}_{2}$ etching under a specific ${\mathrm{CF}}_{4}$/Ar chemistry, and the model’s applicability to other materials (e.g., Si and SiN) or alternative chemistries (e.g., ${\mathrm{Cl}}_{2}$- or HBr-based processes) has not been validated. Third, the generalization study addresses only the geometric transferability across unseen initial critical dimensions under fixed process conditions, and does not cover variations in aspect ratio, mask materials, or chamber configurations. Addressing these limitations through expanded experimental datasets, fine-tuning strategies for domain adaptation, and multi-chemistry TCAD campaigns represent important directions for future work.

## Figures and Tables

**Table 1:** Process parameters and ranges used in TCAD simulations.

Parameter	Range	Samples	Nominal Value	Unit
*P*	[3,25]	1	22	mTorr
$P_{top}$	[500,1500]	25	1000	W
$P_{bot}$	[10,130]	10	120	W
$Q_{Ar}$	[30,60]	5	30	SCCM
$Q_{CF_{4}}$	[10,30]	5	20	SCCM

**Table 2:** Sample parameters for interpretability study.

Samp.	Variant	*p* (mTorr)	$P_{\mathrm{top}}$ (W)	$P_{\mathrm{bot}}$ (W)	$Q_{\mathrm{Ar}}$ (sccm)	$Q_{{\mathrm{CF}}_{4}}$ (sccm)
1	$P_{\mathrm{bot}}$ increased	10	1125	100	40	15
2	Baseline condition	10	1125	60	40	15
3	$Q_{{\mathrm{CF}}_{4}}$ doubled	10	1125	60	40	30
4	$Q_{\mathrm{Ar}}$ increased	10	1125	60	50	15
5	$P_{\mathrm{top}}$ reduced	10	500	60	40	15
6	Pressure elevated	25	1125	60	40	15

**Table 3:** Quantitative comparison of spatio-temporal prediction methods. ↓ indicates a lower value is better, ↑ indicates a higher value is better, and **Bold values** indicate the best performance for each metric.

Method	1000 × MSE ↓	SSIM ↑	PSNR ↑	EDE ↓ (px)	SAE ↓ (°)	BCDE ↓ (px)	Time ↓ (ms)	FPS ↑
ConvLSTM	1.443	0.988	29.001	8.40	**1.27**	7.31	174.52	22.92
PredRNN	4.938	0.962	25.480	13.51	2.57	10.91	100.21	39.91
PhyDNet	1.935	0.987	28.459	**7.79**	1.32	7.80	315.83	12.66
SimVP	1.378	0.990	29.535	8.29	1.53	7.97	159.03	25.15
SimVPv2	1.435	0.988	29.198	7.93	1.46	**7.28**	263.71	13.14
EPreNet	**1.157**	**0.992**	**30.323**	8.28	1.40	7.56	**38.92**	**102.78**

**Table 4:** Ablation study results.

No.	Method	1000 × MSE ↓	SSIM ↑	PSNR ↑	EDE ↓ (px)	SAE ↓ (°)	BCDE ↓ (px)
(a)	w/o Attention	1.557	0.987	28.907	8.21	1.07	7.41
(b)	w/o CFM	1.509	0.987	29.010	8.12	1.30	7.50
(c)	w/o LSTM	1.494	0.971	23.040	14.98	1.23	12.91
(d)	w/o CTA	1.445	0.987	29.220	8.78	1.31	7.45
(e)	BCE only	1.608	0.986	28.563	7.59	1.46	7.38
(f)	MSE + SSIM	1.279	0.991	29.834	7.80	1.42	7.30
(g)	EPreNet	1.157	0.992	30.323	8.28	1.40	7.56

**Table 5:** Results of different input frame lengths.

T	1000 × MSE ↓	SSIM ↑	PSNR ↑	EDE ↓ (px)	SAE ↓ (°)	BCDE ↓ (px)
1	3.065	0.988	27.426	7.07	2.97	7.99
2	2.262	0.990	28.462	6.74	2.61	8.65
5	1.374	0.992	29.945	7.17	1.76	8.25
10	1.157	0.992	30.323	8.28	1.40	7.56

**Table 6:** EPreNet of performance on unseen initial CD configurations (TCAD simulation).

CD	1000 × MSE ↓	SSIM ↑	PSNR ↑	EDE ↓ (px)	SAE ↓ (°)	BCDE ↓ (px)
0.1	2.20	0.9851	27.69	6.00	15.69	1.95
0.2	2.57	0.9838	27.01	10.70	9.50	5.41
0.3	2.84	0.9835	25.94	5.20	9.25	4.07
0.4	3.14	0.9830	25.56	6.40	8.91	4.16
0.5	2.68	0.9841	26.26	9.00	8.86	1.30
0.6	2.29	0.9848	26.84	9.50	2.66	1.19
0.7	2.91	0.9842	25.77	8.10	3.04	2.91
0.8	1.92	0.9865	27.60	9.60	2.96	2.81
0.9	3.16	0.9833	25.51	9.50	2.52	2.90
Average	2.63	0.9843	26.46	8.22	7.07	2.40

**Table 7:** EPreNet of performance on experimental SEM images (no fine-tuning).

Condition	1000 × MSE ↓	SSIM ↑	PSNR ↑	EDE ↓ (px)	SAE ↓ (°)	BCDE ↓ (px)
(i) $P_{\mathrm{top}}=1000$ W	0.0387	0.936	14.45	20.6	8.66	37.8
(ii) $P_{\mathrm{top}}=500$ W	0.0474	0.931	13.65	13.2	6.02	12.4

## Data Availability

Data and code are available at https://github.com/AaHa123/EPreNet (accessed on 27 November 2025).
